# Single-cell transcriptomic characterization of a human MDS hematopoietic dataset and in vitro validation of apoptosis-related venetoclax resistance in human MDS-L cells

**DOI:** 10.3389/fcell.2026.1947721

**Published:** 2026-09-18

**Authors:** Tianjiao Huang, Shiyu Li, Hong Zhou, Heng Guo, Suliang Wang

**Affiliations:** 1 Department of Hematology, Qiqihar Medical University (The Second Affiliated Hospital), Qiqihar, Heilongjiang, China; 2 Clinical Pathology Professional College, General Graduate School of Kyungwoon University, Gumi, North Gyeongsang, Republic of Korea

**Keywords:** apoptosis, ferroptosis, MDS-L cells, myelodysplastic syndromes, single-cell RNA sequencing, venetoclax resistance

## Abstract

**Background:**

Myelodysplastic syndromes (MDS) are heterogeneous clonal hematopoietic disorders in which dysregulated apoptosis, oxidative stress, and adaptive survival signaling contribute to disease persistence and therapeutic resistance. Venetoclax (VEN) targets BCL-2-dependent survival, but resistance is frequently accompanied by compensatory survival programs. Whether ferroptosis-associated transcriptional states are dynamically remodeled during VEN treatment and participate in this adaptive phenotype remains incompletely understood.

**Methods:**

We analyzed two independent human MDS single-cell RNA-sequencing cohorts together with an *in vitro* VEN-resistant MDS-L model. GSE241417 was used to characterize longitudinal and cell-state-specific ferroptosis-associated transcriptional patterns, whereas GSE180298 provided independent single-cell validation. Longitudinal module scores were summarized across major hematopoietic states and treatment stages. Apoptosis/proliferation- and ferroptosis-related genes were further examined by qRT-PCR in parental and VEN-resistant MDS-L cells.

**Results:**

Single-cell analysis revealed marked heterogeneity of ferroptosis-associated transcriptional programs across MDS hematopoietic populations. Longitudinal trajectories in GSE241417 demonstrated nonuniform, treatment-stage- and cell-state-dependent remodeling of ferroptosis balance, inflammatory context, and survival-associated programs. The independent GSE180298 cohort reproduced major disease-associated ferroptosis-related transcriptional differences. In VEN-resistant MDS-L cells, *MCL1*, *BCL2L1*, *MKI67*, *GPX4*, *SLC7A11*, and *NFE2L2* were increased, whereas *BCL2*, *DDIT3*, *ACSL4*, and *NCOA4* were decreased, indicating coordinated adaptation of apoptotic and ferroptosis-related pathways.

**Conclusion:**

This study reveals cell-state-dependent and temporally dynamic ferroptosis-associated remodeling in MDS and identifies a coordinated apoptosis-redox transcriptional program associated with VEN resistance. The integration of longitudinal single-cell analysis, independent single-cell validation, and targeted qRT-PCR supports ferroptosis-defense pathways as a biologically relevant component of VEN-resistant adaptation and provides a rationale for future combination strategies that address both apoptotic dependence and redox-state plasticity.

## 1 Introduction

Myelodysplastic syndromes (MDS) are clonal hematopoietic disorders characterized by ineffective hematopoiesis, peripheral cytopenias, dysplastic morphology, and a variable risk of progression to acute myeloid leukemia (AML) (Cazzola, 2020; Platzbecker, 2019). MDS is biologically heterogeneous, with abnormal stem/progenitor populations coexisting with myeloid, erythroid, and lymphoid cell states. In addition to genetic and epigenetic abnormalities, altered cell-death regulation, oxidative stress, and metabolic adaptation can influence clonal persistence and treatment response.

Venetoclax (VEN), a selective BCL-2 inhibitor, promotes mitochondrial apoptosis in susceptible malignant cells. VEN-based combinations have demonstrated clinically meaningful activity in higher-risk MDS and AML (Garcia et al., 2025; DiNardo et al., 2020; Garcia-Manero, 2023), establishing BCL-2 dependence as a therapeutically actionable vulnerability. Nevertheless, primary and acquired resistance remain major challenges (Bose et al., 2017). Reduced dependence on BCL-2 and compensatory activation of alternative survival programs, particularly MCL1- and BCL2L1-related signaling, may allow malignant cells to persist despite VEN exposure. These observations suggest that VEN resistance is not restricted to a single anti-apoptotic switch but may involve broader remodeling of apoptosis, cellular stress, metabolism, and redox homeostasis.

Ferroptosis is an iron-dependent form of regulated cell death driven by lipid peroxidation and governed by iron metabolism, glutathione homeostasis, and antioxidant defense (Dixon et al., 2012; Stockwell et al., 2017; Jiang et al., 2021). Key regulators such as GPX4, SLC7A11, NFE2L2, ACSL4, TFRC, NCOA4, FTH1, and FTL participate in maintaining the balance between ferroptosis susceptibility and defense. Because apoptotic signaling and redox homeostasis are closely connected to cellular survival, ferroptosis-related adaptation may represent an additional mechanism by which MDS cells tolerate therapeutic stress and acquire resistance to VEN.

Single-cell RNA sequencing (scRNA-seq) enables the resolution of heterogeneous hematopoietic cell states that are obscured in bulk analyses (Stuart and Satija, 2019; Stuart et al., 2019; Korsunsky et al., 2019). Recent MDS studies have shown that stem/progenitor architecture and cell-state composition can influence disease progression and response to venetoclax-based therapy (Ganan-Gomez et al., 2022). However, the distribution and longitudinal remodeling of ferroptosis-associated programs during treatment remain insufficiently characterized. Integrating treatment-stage-resolved single-cell data with an independent single-cell cohort and experimental validation may therefore reveal an additional layer of adaptive biology that complements conventional apoptosis-centered models of VEN resistance.

In this study, we integrated multi-patient scRNA-seq data with an independent human MDS single-cell cohort and a VEN-resistant human MDS-L cell model to characterize ferroptosis-associated transcriptional heterogeneity and its temporal remodeling. We further examined whether apoptosis/proliferation-related changes are accompanied by coordinated alterations in key ferroptosis-regulatory genes during the acquisition of VEN resistance. By linking cell-state-resolved longitudinal transcriptomic features with independent cohort replication and *in vitro* validation, this study provides a multidimensional framework for understanding the interplay between apoptotic adaptation and ferroptosis defense in MDS.

## 2 Materials and methods

### 2.1 Public datasets and study design

The study integrated two publicly available human MDS single-cell transcriptomic datasets. The discovery cohort GSE241417 (GEO, GSE241417) contained 18 bone-marrow scRNA-seq libraries from three MDS patients (UPN1–UPN3) sampled across treatment cycles or stages. The independent cohort GSE180298 (GEO, GSE180298) contained 12 bone-marrow libraries, including five young healthy controls, three elderly healthy controls, and four MDS samples.

GSE241417 was used to characterize longitudinal and cell-state-specific transcriptional changes, whereas GSE180298 was used to assess the reproducibility of major hematopoietic and ferroptosis-associated patterns in an independent cohort. Accession information and sample composition were verified using the corresponding GEO records before analysis.

### 2.2 Single-cell quality control and preprocessing

Raw HDF5 matrices were read as sparse count matrices. Cells were retained when total counts were at least 500, at least 200 genes were detected, and the mitochondrial transcript fraction was no more than 50%. Counts were normalized to a library size of 10,000 and transformed with log1p. GSE180298 features were aligned by shared gene symbols across libraries before variance estimation because the source matrices did not contain identical feature counts. The QC thresholds were applied consistently within each cohort, and retained-cell counts were summarized by library rather than treating cells as independent samples.

### 2.3 Dimensionality reduction, integration, clustering, and cell-state annotation

For each cohort, the 2,000 most variable shared genes were selected. The normalized expression matrix was scaled, and principal-component analysis was performed using 50 components. Harmony integration was applied using library identity as the batch variable with a fixed random seed of 42 and a maximum of 10 iterations. A 30-nearest-neighbor graph was constructed from the Harmony-corrected representation, followed by Leiden clustering at a resolution of 0.8. UMAP visualization was generated from the first 30 Harmony-corrected components using 30 neighbors, Euclidean distance, and a min_dist value of 0.30. For visualization, up to 500 quality-controlled cells per library were sampled with the same random seed, whereas all retained cells were used for quantitative analyses. Broad cell states were annotated according to established human hematopoietic lineage-associated expression patterns; HSPC-like cells were supported by expression of CD34, SPINK2, GATA2, KIT, HLF, and MEIS1.

### 2.4 Ferroptosis-related gene sets and score calculation

The prespecified compact framework (Table 1) comprised a susceptibility module (*ACSL4*, *TFRC*, *NCOA4*, *SAT1*, and *LPCAT3*) and a defense module (*GPX4*, *SLC7A11*, *NFE2L2*, *GCLC*, *GSS*, *AIFM2*, *FTH1*, and *FTL*). For each cell, the ferroptosis-associated balance was defined as susceptibility score minus defense score using log1p-normalized expression. For longitudinal visualization, an apoptosis/survival contextual score was derived from the BCL-2-family genes represented in the bridge framework, while the *TNF*–NF-κB and bridge modules were retained as complementary contextual measures; the bridge module consisted of *BCL2*, *MCL1*, *BCL2L1*, *DDIT3*, and *MKI67*. Mean module scores were summarized across HSPC-like, myeloid, monocyte, and granulocytic states at treatment stages C0, C1, C2, C4, C7, C8, and C12. Pairwise gene relationships, cluster-level expression summaries, and ferroptosis-associated score distributions were visualized descriptively across cell states.

**TABLE 1:** Prespecified gene modules used for ferroptosis-associated and longitudinal contextual scoring.

Module	Genes or definition
Susceptibility	*ACSL4*; *TFRC*; *NCOA4*; *SAT1*; *LPCAT3*
Defense	*GPX4*; *SLC7A11*; *NFE2L2*; *GCLC*; *GSS*; *AIFM2*; *FTH1*; *FTL*
Balance	Susceptibility score − defense score
Apoptosis/survival	*BCL2*; *MCL1*; *BCL2L1*
*TNF*–NF-κB	*TNFRSF1A*; *NFKB1*; *RELA*; *NFKBIA*; *TNFAIP3*; *BIRC3*; *TNF*
Bridge	*BCL2*; *MCL1*; *BCL2L1*; *DDIT3*; *MKI67*

### 2.5 Ferroptosis-high/low comparison

High and low ferroptosis-associated states were defined within the same library and annotated cell state using the top and bottom ferroptosis-balance quartiles, respectively. Ranked-gene patterns, UMAP distributions, cluster composition, and expression of representative ferroptosis-related genes were compared descriptively between the two states.

### 2.6 MDS-L cell culture and qRT-PCR

The human MDS-L cell line was obtained from the RIKEN Cell Bank (Tsukuba, Japan) and cultured in RPMI-1640 medium supplemented with 10% fetal bovine serum, 1% penicillin–streptomycin, and 10 ng/mL recombinant human IL-3 at 37 °C in a humidified 5% CO_2_ incubator. To generate a venetoclax-resistant derivative, parental MDS-L cells were continuously exposed to stepwise escalating concentrations of venetoclax (ABT-199), beginning at 10 nM and increasing to 1 μM over approximately 10–12 weeks. Cells maintaining stable growth at 1 μM venetoclax were designated MDS-L VEN-R, whereas untreated parental cells served as controls. Total RNA was extracted using TRIzol and reverse-transcribed into cDNA. qRT-PCR was performed for the apoptosis/proliferation-related genes *BCL2*, *MCL1*, *BCL2L1*, *DDIT3*, and *MKI67* and the ferroptosis-associated genes *GPX4*, *SLC7A11*, *ACSL4*, *NCOA4*, and *NFE2L2*. Expression was normalized to *GAPDH*, and relative fold change was calculated using the 2^−ΔΔCT^ method. Each assay included technical triplicates, and the plotted data represent three independent biological experiments. The primer sequences used for qRT-PCR are provided in Supplementary Table S1.

### 2.7 Statistical analysis

Single-cell analyses were summarized descriptively at the sample/library and annotated cell-state levels, thereby avoiding the treatment of individual cells as independent biological replicates. Longitudinal changes in UPN1 were described across treatment stages. qRT-PCR data from three independent biological experiments are presented as mean ± standard deviation, and parental and VEN-R groups were compared using a two-tailed unpaired Student’s t-test. A two-tailed P value <0.05 was considered statistically significant.

## 3 Results

### 3.1 Single-cell transcriptomic profiling reveals heterogeneous cellular states in MDS bone marrow

After quality control, 93,519 cells from 18 GSE241417 libraries were retained for analysis. Principal-component analysis, Harmony integration, and Leiden clustering resolved 26 transcriptionally distinct clusters. Quality-control distributions, principal-component structure, and UMAP visualization demonstrated substantial cellular heterogeneity across the integrated bone-marrow dataset (Figures 1A–H). Importantly, total counts and mitochondrial transcript percentages were distributed across the UMAP rather than being confined to individual clusters, indicating that the principal cellular structure was not simply driven by obvious quality-control gradients. The library-balanced visualization subset preserved the major cluster architecture while all QC-passed cells were retained for quantitative analyses.

**FIGURE 1:**
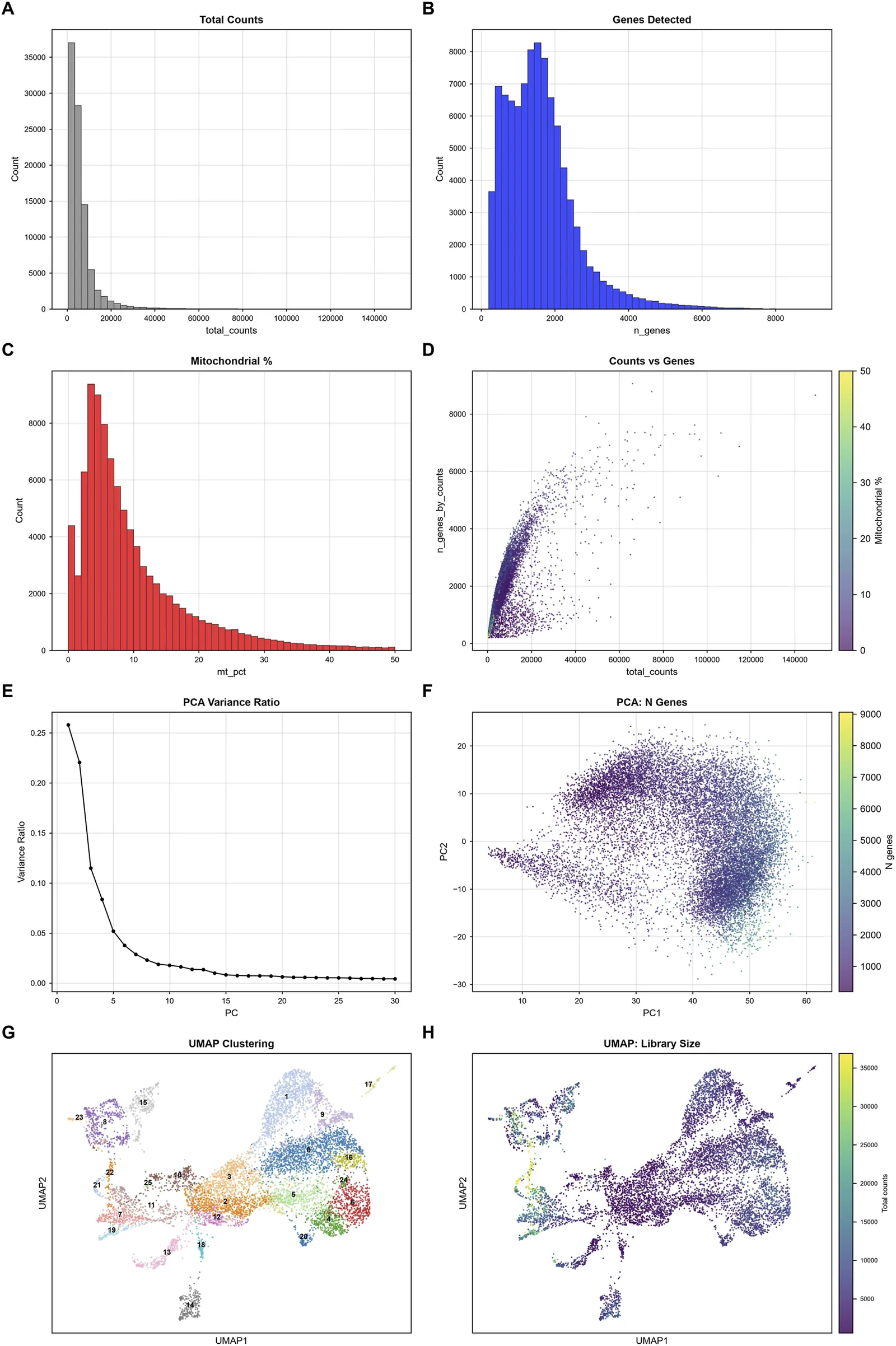
Cell-level quality control, principal components, and formal integration in GSE241417. Histograms show **(A)** total counts, **(B)** detected genes, and **(C)** mitochondrial percentage. **(D)** Total counts versus detected genes, coloured by mitochondrial percentage. **(E)** Variance ratio across the first 30 saved principal components. **(F)** PC1 versus PC2 coloured by detected genes. **(G)** Real single-cell Harmony UMAP coloured and labelled by Leiden cluster. **(H)** The identical UMAP coordinates coloured by total counts. UMAP panels use a reproducible library-balanced subset of 9,000 cells; quantitative analyses use all 93,519 QC-passed cells.

### 3.2 Ferroptosis-associated transcriptional landscape across MDS cell states

Ferroptosis-associated balance scores displayed marked variation across hematopoietic cell states in the integrated GSE241417 dataset. UMAP mapping showed that ferroptosis-related transcriptional activity was unevenly distributed among clusters rather than uniformly expressed across the bone-marrow compartment. Key genes including *GPX4*, *SLC7A11*, *ACSL4*, *TFRC*, *FTH1*, and *TP53* exhibited distinct spatial expression patterns (Figures 2A–H). *FTH1* showed relatively broad expression, whereas several susceptibility- and defense-associated genes were concentrated in more restricted cellular regions. These patterns indicate that individual MDS hematopoietic populations differ not only in lineage identity but also in the balance between ferroptosis susceptibility and antioxidant defense capacity.

**FIGURE 2:**
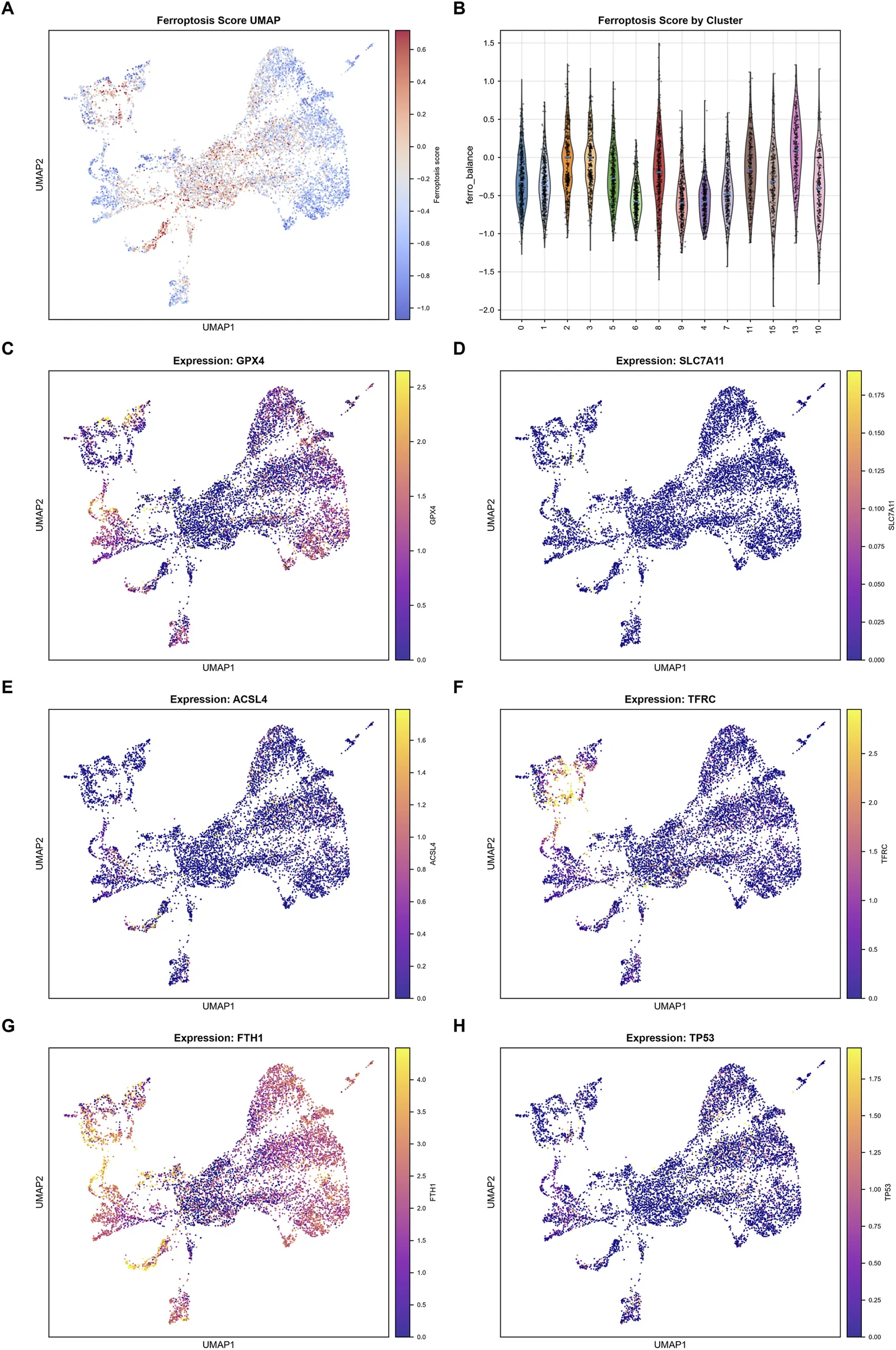
Ferroptosis-associated balance and gene expression on real GSE241417 single-cell coordinates. **(A)** Compact susceptibility-minus-defense balance on the Harmony UMAP. **(B)** Cell-level balance distributions in the 14 most abundant Leiden clusters. The identical UMAP coordinates show log1p-normalized expression of **(C)** *GPX4*, **(D)** *SLC7A11*, **(E)** *ACSL4*, **(F)** *TFRC*, **(G)** *FTH1*, and **(H)** *TP53*. Continuous colours are clipped at the first and 99th percentiles for visualization.

### 3.3 Cluster-specific expression patterns of ferroptosis-related genes

Violin-plot analysis further demonstrated cluster-dependent expression of *GPX4*, *SLC7A11*, *ACSL4*, *TFRC*, *FTH1*, *NCOA4*, *HMOX1*, and *SAT1*. *FTH1* was broadly detected across multiple populations, whereas *SLC7A11*, *ACSL4*, *NCOA4*, *HMOX1*, and *SAT1* showed more restricted or cell-state-enriched expression (Figures 3A–H). Pairwise expression and density analyses showed that *GPX4*–*SLC7A11* and *ACSL4*–*TFRC* relationships were not uniformly coupled across clusters, while the distributions of ferroptosis balance and additional glutathione- and mitochondrial-associated genes such as *GSS*, *GCLC*, *VDAC2*, and *CISD1* further separated redox-associated cellular states (Figures 4A–H). Collectively, these analyses support the presence of multiple ferroptosis-associated transcriptional configurations within the MDS bone-marrow microenvironment rather than a single global ferroptosis phenotype.

**FIGURE 3:**
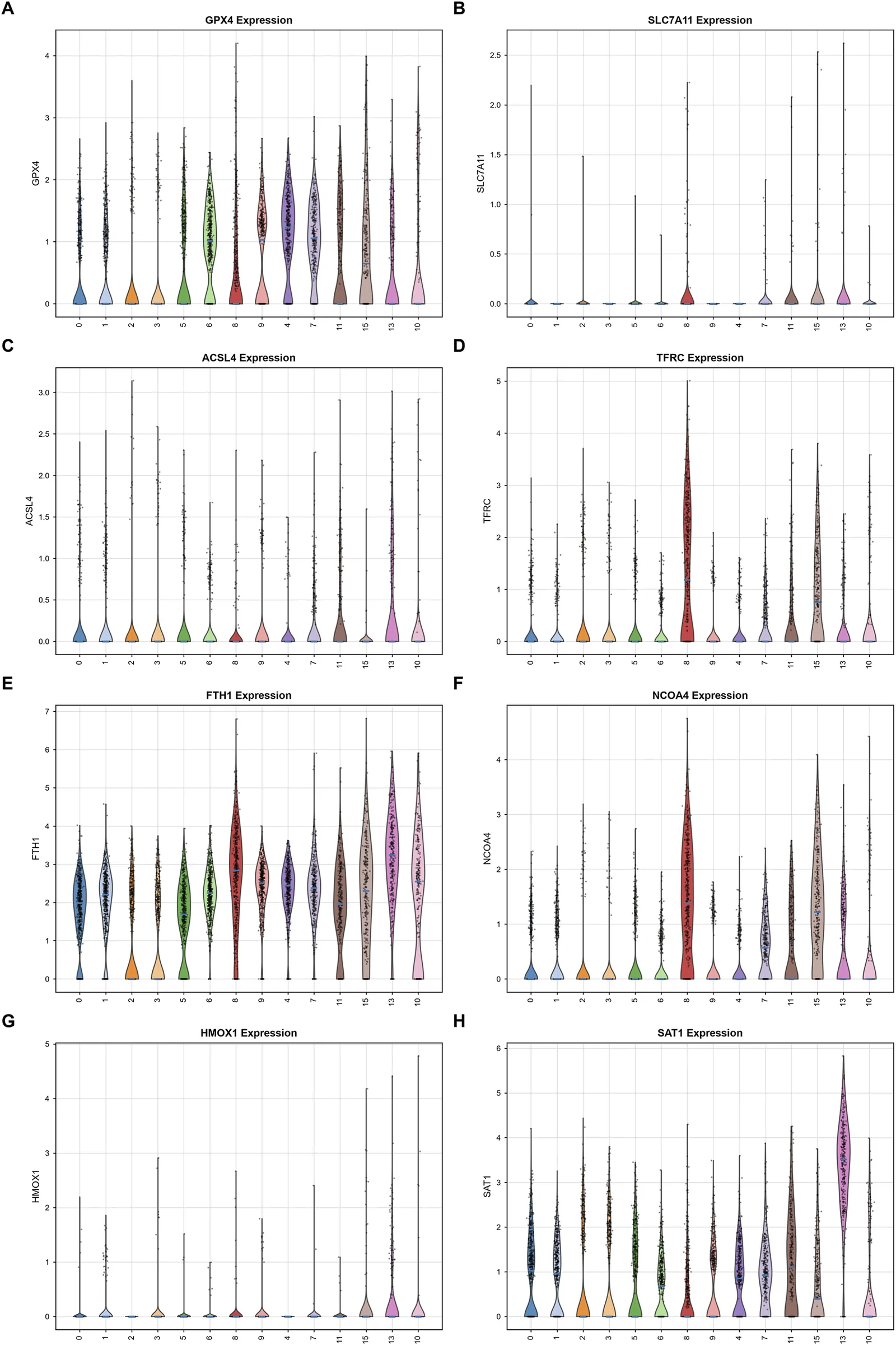
Cluster-level expression distributions in GSE241417. Cell-level violin plots for the 14 most abundant Leiden clusters show log1p-normalized expression of **(A)** *GPX4*, **(B)** *SLC7A11*, **(C)** *ACSL4*, **(D)** *TFRC*, **(E)** *FTH1*, **(F)** *NCOA4*, **(G)** *HMOX1*, and **(H)** *SAT1*. Black points are observed cells in the library-balanced display subset.

**FIGURE 4:**
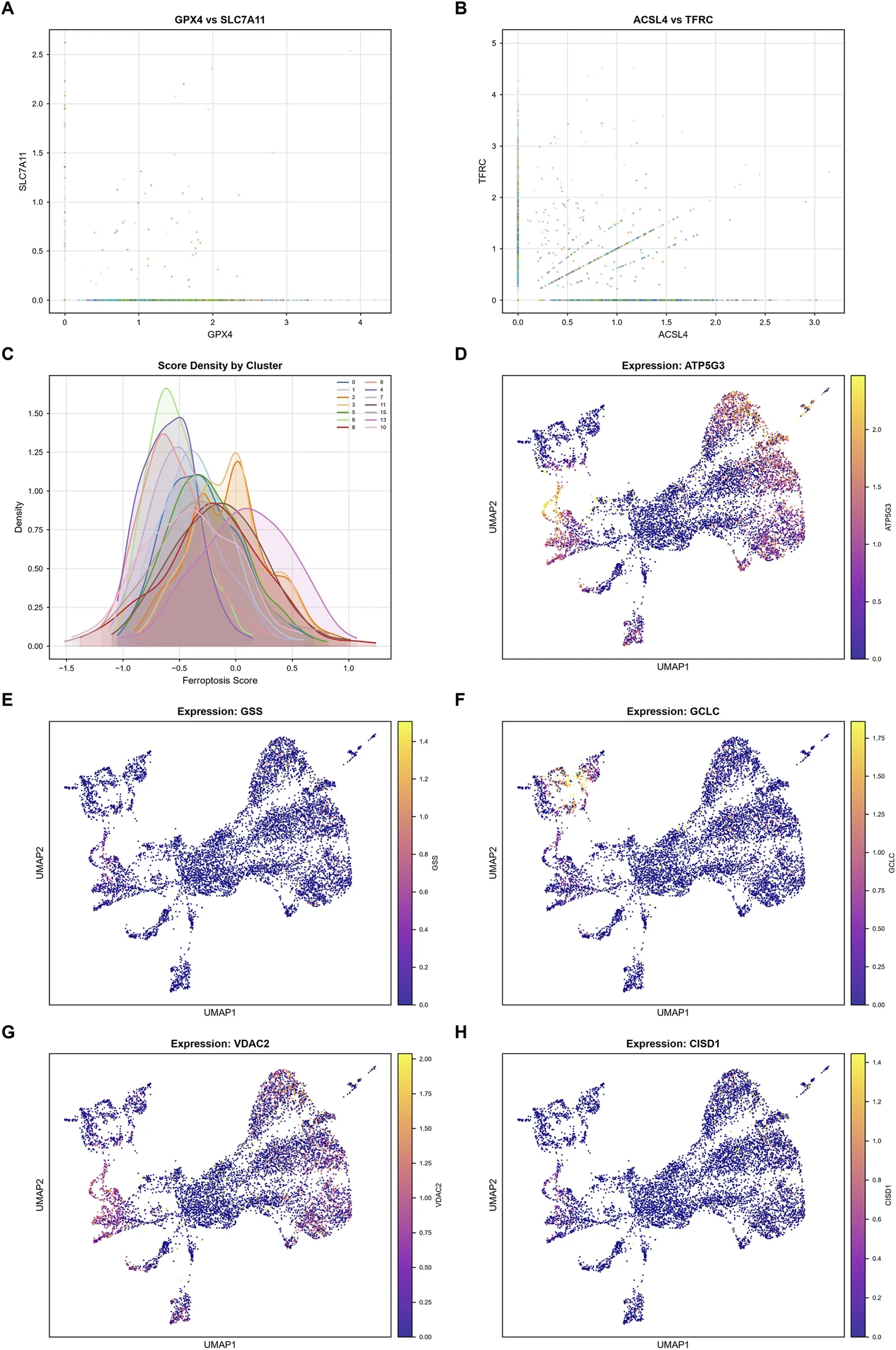
Pairwise and UMAP expression structure in GSE241417. Cell-level scatterplots compare **(A)** *GPX4* with *SLC7A11* and **(B)** *ACSL4* with *TFRC*, coloured by Leiden cluster. **(C)** Kernel-density curves show the compact balance by the 14 most abundant clusters. Real single-cell Harmony UMAPs show log1p-normalized expression of **(D)** *ATP5G3*/*ATP5MC3*, **(E)** *GSS*, **(F)** *GCLC*, **(G)** *VDAC2*, and **(H)** *CISD1*.

### 3.4 Longitudinal remodeling of ferroptosis-associated programs during venetoclax treatment

Longitudinal analysis of UPN1 across C0, C1, C2, C4, C7, C8, and C12 revealed dynamic and non-monotonic remodeling of ferroptosis-, inflammatory-, and survival-associated programs (Figures 5A–D). In HSPC-like cells, the ferroptosis-associated balance changed from −0.240 at C0 to −0.404 at C7, followed by partial recovery to −0.262 at C8 and −0.276 at C12. The corresponding *TNF*–NF-κB scores were 0.492, 0.250, 0.373, and 0.386, and bridge-module scores were 0.390, 0.150, 0.329, and 0.303, respectively, indicating a marked intermediate-stage trough with subsequent rebound. Monocytes showed a reduction in the *TNF*–NF-κB score from 0.920 at C0 to 0.636 at C12, accompanied by a shift in ferroptosis-associated balance from +0.036 to −0.138; the trajectory included negative intermediate stages and a transient rebound around C7. Myeloid and granulocytic populations displayed distinct intermediate-stage fluctuations in ferroptosis balance, apoptosis/survival context, and bridge-gene scores. Thus, VEN treatment was associated with cell-state-specific temporal trajectories rather than a uniform monotonic shift, emphasizing the adaptive plasticity of redox and survival programs during therapy.

**FIGURE 5:**
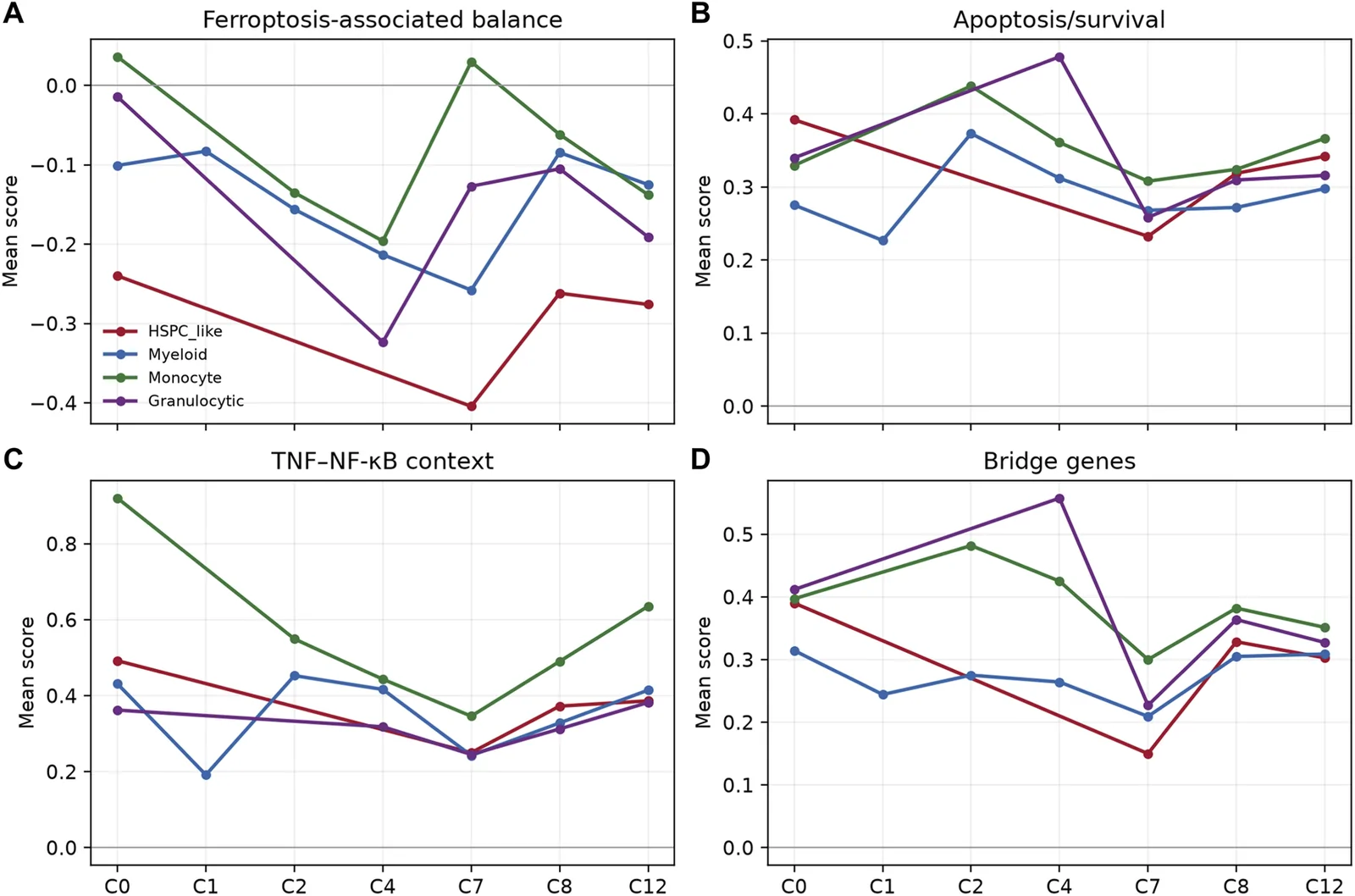
Longitudinal cell-state-resolved remodeling of ferroptosis- and survival-associated programs in UPN1 during venetoclax treatment. Mean module scores are shown across treatment stages C0, C1, C2, C4, C7, C8, and C12 for HSPC-like, myeloid, monocyte, and granulocytic states. **(A)** Ferroptosis-associated balance, calculated as susceptibility minus defense score. **(B)** Apoptosis/survival contextual score. **(C)** *TNF*–NF-κB contextual score. **(D)** Bridge-gene score integrating apoptosis/proliferation-associated transcripts. The trajectories demonstrate nonuniform, stage- and cell-state-dependent remodeling across treatment.

### 3.5 Identification of ferroptosis-high and ferroptosis-low cellular populations

To further characterize ferroptosis-related heterogeneity, cells were stratified into ferroptosis-high and ferroptosis-low states using the top and bottom balance-score quartiles within the same library and annotated cell type. This within-library, within-state design minimized confounding by inter-sample and lineage-composition differences. Ranked-gene analysis, UMAP mapping, and cluster-composition plots demonstrated distinct transcriptional distributions between the two extreme states. *GPX4*, *NFE2L2*, *HSPB1*, and *FTH1* also showed different expression patterns between ferroptosis-high and ferroptosis-low cells, further supporting the presence of reproducible redox-associated cellular phenotypes across multiple MDS hematopoietic compartments (Figures 6A–H).

**FIGURE 6:**
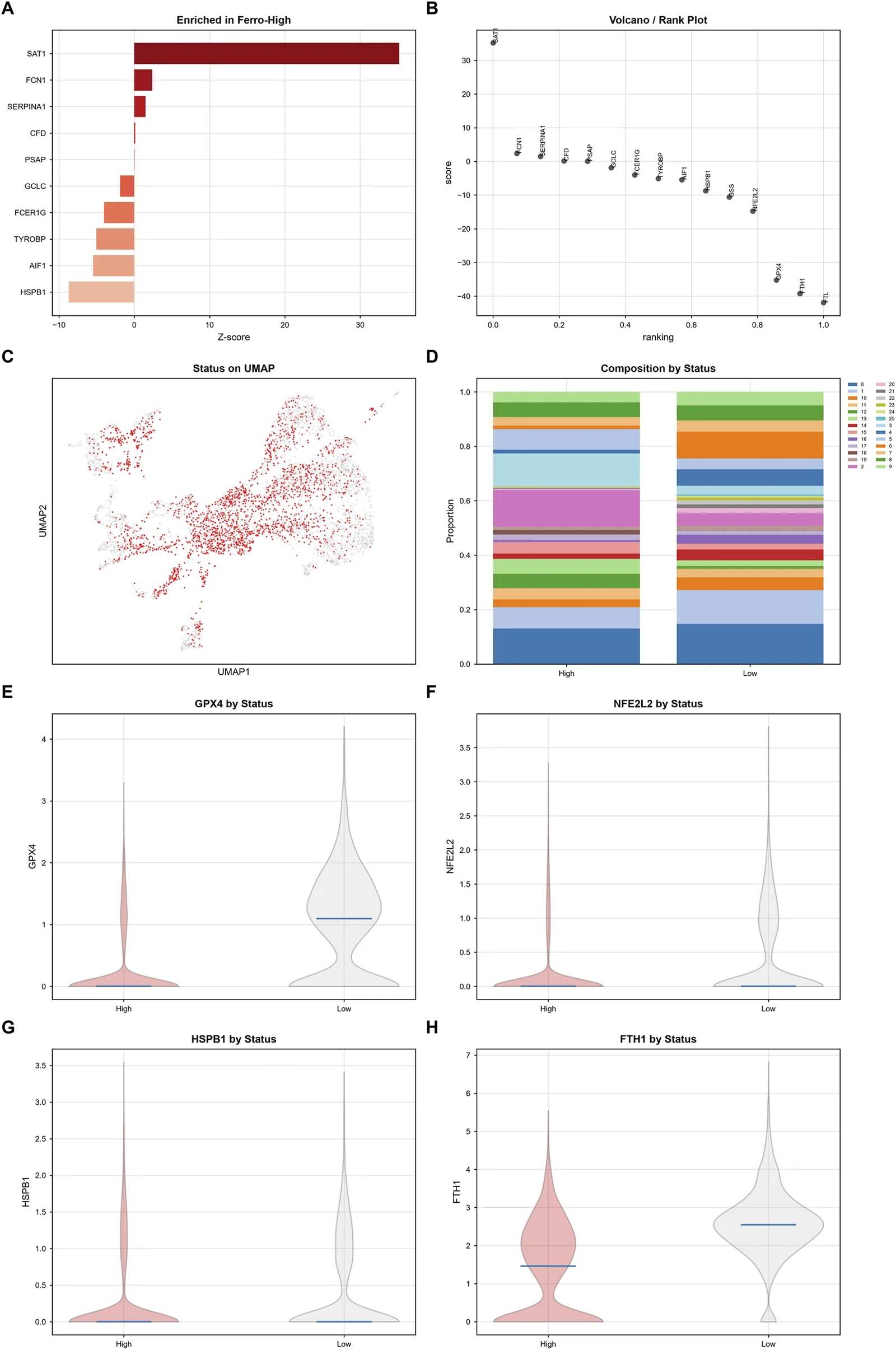
Within-library, within-cell-state high-versus-low contrasts in GSE241417. High and low denote the top and bottom compact-balance quartiles within the same library and annotated cell state; middle-quartile cells are shown only as a pale background in **(C)** and are excluded from contrasts. **(A)** Ranked gene Z-scores and **(B)** the corresponding rank plot. **(C)** Extreme-score cells on the real Harmony UMAP. **(D)** Leiden-cluster composition of high and low strata. Cell-level violins compare **(E)** *GPX4*, **(F)** *NFE2L2*, **(G)** *HSPB1*, and **(H)** *FTH1*.

### 3.6 Independent cohort validation of hematopoietic and ferroptosis-associated features

Analysis of the independent GSE180298 cohort reproduced the expected stem/progenitor, myeloid, T-cell, and NK-cell transcriptional architecture based on lineage-associated marker expression (Figures 7A–H). Ferroptosis-balance distributions, empirical cumulative distributions, *GPX4* density, selected gene-expression differences, and additional cluster-level summaries further demonstrated disease-associated variation in ferroptosis-related transcriptional features between MDS and elderly healthy marrow (Figures 8A–H). The recurrence of both hematopoietic structure and redox-associated differences in a separate cohort strengthens the generalizability of the central single-cell observation: ferroptosis-related transcriptional heterogeneity is a reproducible feature of MDS marrow rather than an isolated property of the discovery dataset.

**FIGURE 7:**
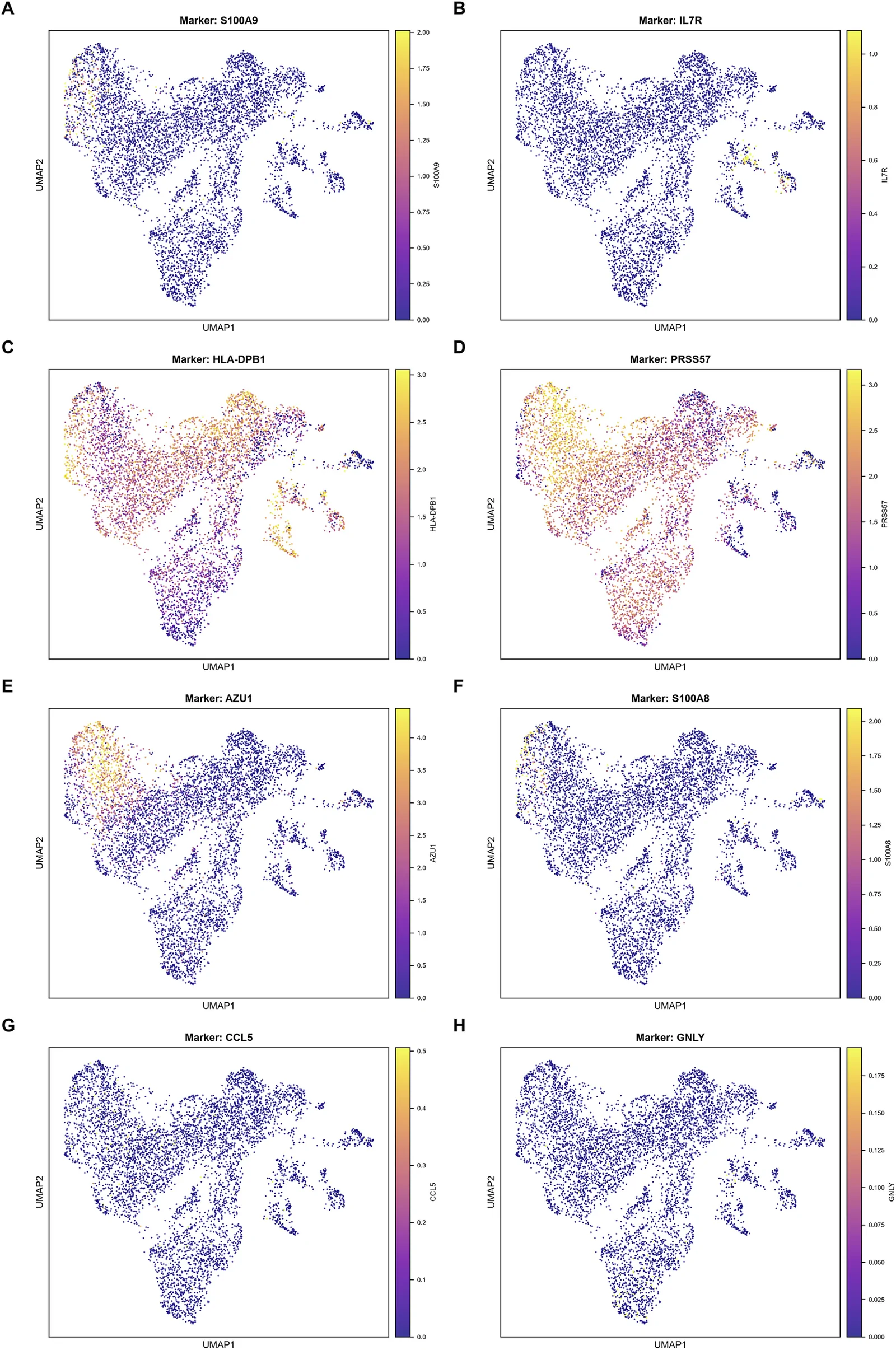
Marker-expression maps in the independent GSE180298 cohort. Real single-cell Harmony UMAPs show log1p-normalized expression of **(A)** S100A9, **(B)** *IL7R*, **(C)** *HLA-DPB1*, **(D)** *PRSS57*, **(E)** *AZU1*, **(F)** S100A8, **(G)** *CCL5*, and **(H)** *GNLY*. All panels use the identical library-balanced coordinates for visualization.

**FIGURE 8:**
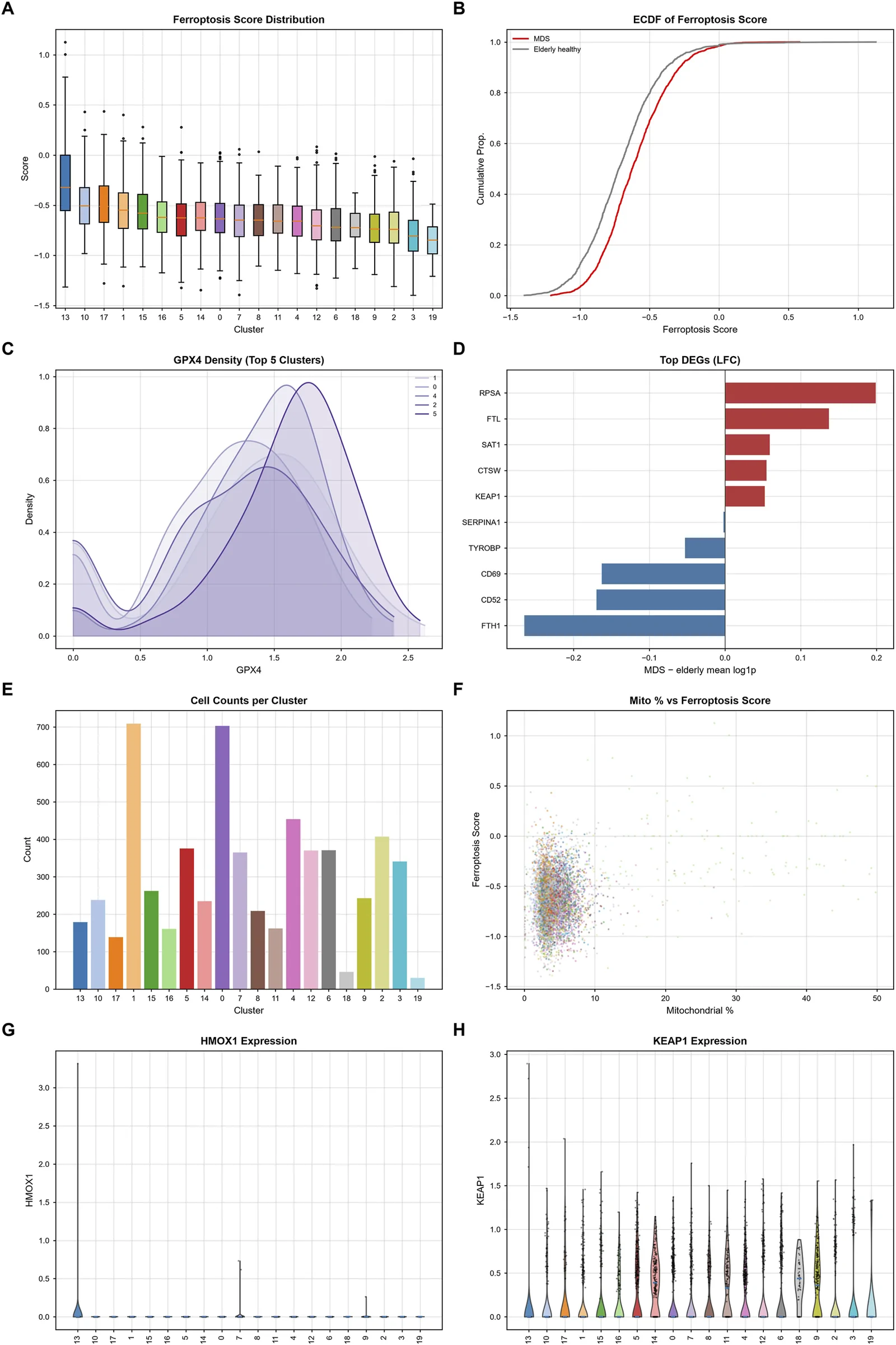
Compact-balance, differential-expression, and quality-control summaries in GSE180298. **(A)** Cell-level balance boxplots ordered by Leiden-cluster median. **(B)** Empirical cumulative distributions in MDS and elderly healthy marrow. **(C)** *GPX4* density in the five most abundant clusters. **(D)** Descriptive sampled-cell mean log1p expression differences for selected genes, MDS minus elderly healthy. **(E)** Display-cell counts by cluster. **(F)** Mitochondrial percentage versus balance. Violin plots show **(G)** *HMOX1* and **(H)** *KEAP1* by cluster. Panels are presented as descriptive summaries of the independent cohort.

### 3.7 Integrated cross-gene and cluster-level characterization of ferroptosis-associated features

Cross-gene and cluster-level analyses further characterized ferroptosis-associated heterogeneity in GSE241417. Gene-correlation patterns, *GPX4*–*FTH1* density, cluster-level expression profiles, ferroptosis-high/low composition, the *SLC7A11*–*GPX4* relationship, mitochondrial characteristics, mean-expression heatmaps, and *TP53* distributions collectively demonstrated that ferroptosis-associated genes are organized differently across MDS hematopoietic states (Figures 9A–H). The lack of a single uniform correlation structure across the analyzed genes further supports the concept that ferroptosis susceptibility and defense emerge from coordinated but context-dependent transcriptional programs rather than from the behavior of an individual marker.

**FIGURE 9:**
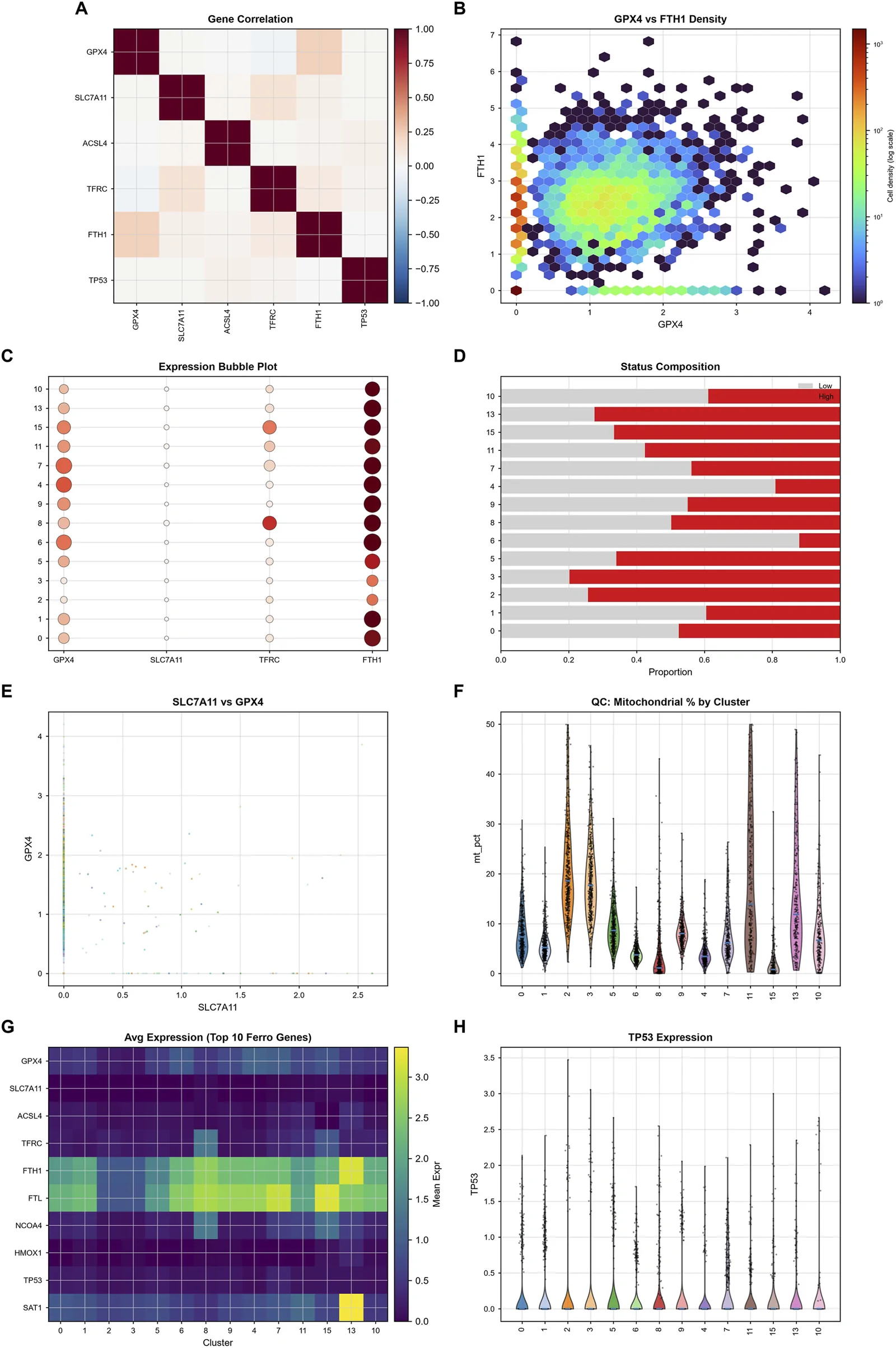
Cross-gene, cluster, status, and quality-control summaries in GSE241417. **(A)** Pearson correlation heatmap. **(B)** *GPX4*–*FTH1* hexagonal density. **(C)** Cluster-level expression bubble plot, with size representing the detected fraction and colour representing mean expression. **(D)** High/low quartile composition within each Leiden cluster after excluding middle-quartile cells. **(E)** *SLC7A11* versus *GPX4* scatterplot coloured by Leiden cluster. **(F)** Mitochondrial-percentage violins. **(G)** Mean-expression heatmap for ten ferroptosis-associated genes. **(H)** *TP53* expression violins.

### 3.8 Validation of apoptosis/proliferation- and ferroptosis-related gene expression in parental and venetoclax-resistant MDS-L cells

To experimentally examine the transcriptional adaptations associated with VEN resistance, apoptosis/proliferation- and ferroptosis-related genes were measured in parental and VEN-resistant MDS-L cells. Compared with parental cells, VEN-R cells showed significantly increased *MCL1*, *BCL2L1*, and *MKI67* expression together with reduced *BCL2* and *DDIT3* expression, consistent with redistribution toward alternative survival signaling, enhanced proliferative activity, and altered cellular-stress responses (Figure 10A). In parallel, *GPX4*, *SLC7A11*, and *NFE2L2* were significantly upregulated, whereas *ACSL4* and *NCOA4* were reduced in VEN-R cells (Figure 10B). The coordinated direction of these changes is notable because it links the established apoptosis-resistance phenotype to a transcriptional shift favoring ferroptosis defense. The qRT-PCR data therefore provide experimental support for the redox-remodeling patterns identified by the single-cell analyses and reinforce the concept of a coupled apoptosis–ferroptosis adaptation during VEN resistance.

**FIGURE 10:**
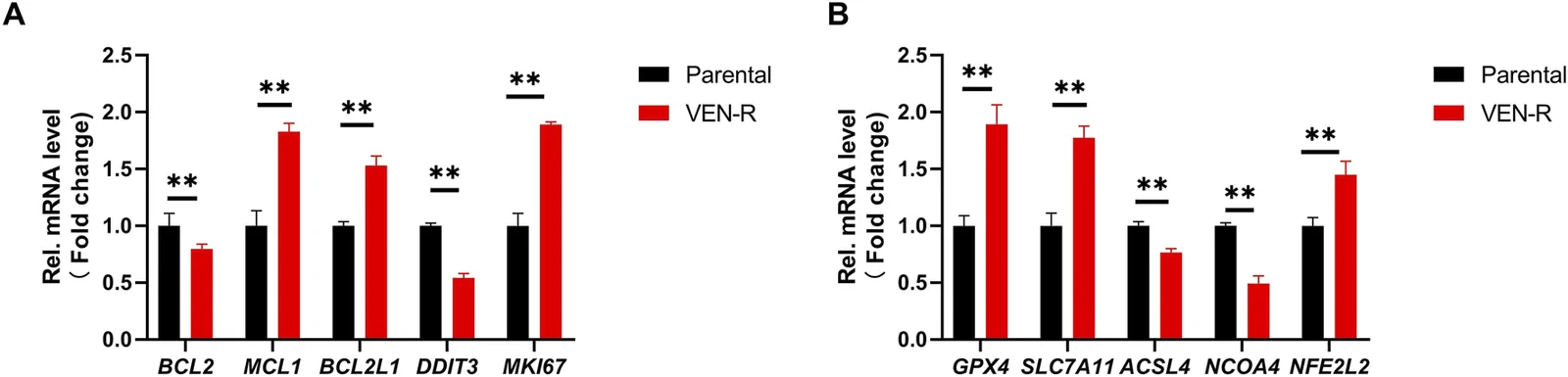
qRT-PCR validation of apoptosis/proliferation and ferroptosis-associated transcriptional remodeling in parental and venetoclax-resistant MDS-L cells. **(A)** Relative mRNA levels of *BCL2*, *MCL1*, *BCL2L1*, *DDIT3*, and *MKI67*. **(B)** Relative mRNA levels of the ferroptosis-associated genes *GPX4*, *SLC7A11*, *ACSL4*, *NCOA4*, and *NFE2L2*. Expression was normalized to *GAPDH* and calculated using the 2^−ΔΔCT^ method, with the parental group set as the reference. VEN-R cells showed increased *MCL1*, *BCL2L1*, *MKI67*, *GPX4*, *SLC7A11*, and *NFE2L2* and decreased *BCL2*, *DDIT3*, *ACSL4*, and *NCOA4*. Data are presented as mean ± SD from three independent biological experiments. **P < 0.01 versus parental cells by two-tailed unpaired Student’s t-test.

## 4 Discussion

The present study integrates longitudinal single-cell transcriptomics, independent single-cell validation, and *in vitro* qRT-PCR to investigate ferroptosis-associated transcriptional remodeling in MDS and its relationship with VEN resistance. Three findings define the central contribution of the work. First, ferroptosis-associated programs are highly cell-state dependent across the MDS marrow compartment. Second, the newly resolved longitudinal trajectories demonstrate that redox, inflammatory, and survival-associated programs are dynamically and nonlinearly remodeled during VEN treatment. Third, VEN-resistant MDS-L cells show coordinated transcriptional changes in both BCL-2-family/proliferation genes and core ferroptosis regulators. Together, these results support an integrated model in which apoptotic adaptation and redox defense evolve in parallel rather than as biologically isolated processes.

MDS is characterized by marked hematopoietic heterogeneity, and increasing evidence indicates that stem/progenitor architecture is biologically relevant to venetoclax response (Ganan-Gomez et al., 2022). Our single-cell analyses extend this concept by showing that ferroptosis-associated genes are also unevenly distributed across hematopoietic states. *GPX4*, *SLC7A11*, *ACSL4*, *TFRC*, *FTH1*, *NCOA4*, *HMOX1*, and *SAT1* displayed distinct cell-state-specific patterns, indicating that redox-state diversity constitutes an additional dimension of MDS heterogeneity. This is important because cells occupying different hematopoietic states may face different oxidative, metabolic, and iron-handling constraints even within the same marrow environment. The present study therefore moves beyond a lineage-only description of heterogeneity and identifies ferroptosis-related transcriptional organization as a potentially treatment-relevant state variable.

The longitudinal analysis adds a particularly informative temporal dimension. In HSPC-like cells, the ferroptosis balance became more defense-skewed by C7 while *TNF*–NF-κB and bridge scores reached an intermediate-stage nadir before partially recovering. Monocytes followed a different trajectory, with an initially positive ferroptosis balance shifting toward a more negative state by C12 alongside persistent attenuation of the inflammatory-context score. Myeloid and granulocytic compartments showed their own intermediate-stage fluctuations. The non-monotonic nature of these trajectories argues for adaptive state transitions rather than a simple uniform pharmacologic effect. This temporal plasticity may help explain why a snapshot taken at a single treatment time point can underestimate the complexity of response and resistance biology.

The MDS-L experiments provide an experimental bridge between these transcriptomic observations and VEN-resistant cellular adaptation. Increased *MCL1* and *BCL2L1* together with reduced *BCL2* are consistent with redistribution away from exclusive BCL-2 dependence, a recognized route to venetoclax resistance in myeloid malignancies (Bose et al., 2017; Zhang et al., 2022; Carter et al., 2022). Increased *MKI67* and reduced *DDIT3* further indicate altered proliferative and stress-response programs. Importantly, these apoptosis-related changes occurred together with increased *GPX4*, *SLC7A11*, and *NFE2L2* and reduced *ACSL4* and *NCOA4*. The direction of this gene-expression pattern is collectively consistent with strengthened ferroptosis defense: *GPX4* and *SLC7A11* support glutathione-dependent control of lipid peroxidation, *NFE2L2* coordinates antioxidant responses, *ACSL4* contributes to the generation of peroxidation-prone phospholipids, and *NCOA4* regulates ferritinophagy and intracellular iron availability (Dixon et al., 2012; Stockwell et al., 2017; Jiang et al., 2021). Thus, the resistant phenotype is characterized not simply by an anti-apoptotic switch but by broader remodeling of cell-death susceptibility.

A key strength and innovation of this study is the convergence of three complementary levels of evidence within one analytical framework: cell-state-resolved longitudinal profiling, replication in an independent MDS single-cell cohort, and targeted validation in a VEN-resistant human MDS-L model. Previous work has established the clinical activity of VEN-based regimens in higher-risk MDS (Garcia et al., 2025; Bazinet et al., 2022) and the importance of stem-cell architecture and alternative anti-apoptotic signaling in treatment response (Ganan-Gomez et al., 2022; Zhang et al., 2022; Carter et al., 2022). The present analysis adds a distinct layer by connecting these established resistance concepts to ferroptosis-associated redox remodeling. Rather than treating apoptosis and ferroptosis as separate endpoints, our data support a coordinated apoptosis–redox adaptation that is observable across treatment-stage trajectories and experimentally reproduced in resistant cells.

The independent GSE180298 analysis further increases the biological relevance of the discovery findings. Reproduction of major hematopoietic structures and disease-associated ferroptosis-related differences across a second cohort reduces the likelihood that the core observation reflects only one library composition or one analytical realization. At the same time, the within-library and within-cell-state high/low comparisons provide an orthogonal view of redox heterogeneity that is less sensitive to differences in cell-type abundance. These complementary analyses make the central conclusion more robust: ferroptosis-associated heterogeneity is embedded within MDS hematopoietic organization and remains detectable across datasets and analytical scales.

Overall, the study offers a biologically coherent extension of current VEN-resistance models. MCL-1-associated escape from BCL-2 inhibition is well established (Bose et al., 2017; Zhang et al., 2022; Carter et al., 2022), but the accompanying redox adaptation has received less attention in the MDS context. By identifying coordinated changes in apoptosis/proliferation and ferroptosis-regulatory genes, and by placing those changes against a longitudinal single-cell background, the present work highlights a potentially actionable interface between two regulated cell-death programs. This integrated perspective may be useful for biomarker development, treatment-timing studies, and rational design of combination strategies in higher-risk MDS.

## 5 Limitations

Several considerations should be noted when interpreting the findings. The two public single-cell cohorts differ in sample composition, and the MDS-L experiments primarily provide transcriptional rather than protein-level or functional ferroptosis evidence. These features define the next experimental steps, including direct lipid-peroxidation, iron-dependence, and rescue assays. Nevertheless, the agreement among longitudinal cell-state trajectories, an independent single-cell cohort, within-state ferroptosis stratification, and the VEN-resistant cell model provides a consistent multi-level basis for the central conclusion and supports further mechanistic evaluation of the apoptosis–ferroptosis interface.

## 6 Conclusion

This study demonstrates that ferroptosis-associated transcriptional programs are heterogeneous across MDS hematopoietic cell states and are dynamically remodeled during VEN treatment. The newly incorporated longitudinal analysis shows distinct temporal trajectories across HSPC-like, myeloid, monocyte, and granulocytic compartments, while an independent single-cell cohort supports the reproducibility of disease-associated redox heterogeneity. qRT-PCR in VEN-resistant MDS-L cells further reveals coordinated upregulation of *MCL1*, *BCL2L1*, *MKI67*, *GPX4*, *SLC7A11*, and *NFE2L2* together with downregulation of *BCL2*, *DDIT3*, *ACSL4*, and *NCOA4*. By integrating temporal single-cell resolution, independent validation, and experimental confirmation, our findings identify a coordinated apoptosis–ferroptosis adaptive program associated with VEN resistance and provide a focused rationale for future therapeutic strategies targeting both apoptotic dependence and ferroptosis-defense pathways in MDS.

## Data Availability

The datasets presented in this study can be found in online repositories. The names of the repository/repositories and accession number(s) can be found in the article/Supplementary Material.
