# Supplementary material for: Single-cell transcriptomic characterization of a human MDS hematopoietic dataset and in vitro validation of apoptosis-related venetoclax resistance in human MDS-L cells
Source: Front Cell Dev Biol. 2026 Sep 18;14:1947721. doi: 10.3389/fcell.2026.1947721 (PMC13630996; doi:10.3389/fcell.2026.1947721)
Supplement: Supplementary file 1 [file Table1.docx]

**Supplementary Table S1. qRT-PCR primer sequences corresponding to the revised manuscript targets**

| **Functional group** | **Gene** | **Forward primer (5′→3′)** | **Reverse primer (5′→3′)** | **RefSeq accession** | **Catalog no.** | **Species** |
| --- | --- | --- | --- | --- | --- | --- |
| Apoptosis / proliferation | *BCL2* | ATCGCCCTGTGGATGACTGAGT | GCCAGGAGAAATCAAACAGAGGC | NM_000633 | HP200598 | Human |
| Apoptosis / proliferation | *MCL1* | CCAAGAAAGCTGCATCGAACCAT | CAGCACATTCCTGATGCCACCT | NM_021960 | HP214280 | Human |
| Apoptosis / proliferation | *BCL2L1* | GCCACTTACCTGAATGACCACC | AACCAGCGGTTGAAGCGTTCCT | NM_138578 | HP234144 | Human |
| Apoptosis / proliferation | *DDIT3* | GGTATGAGGACCTGCAAGAGGT | CTTGTGACCTCTGCTGGTTCTG | NM_004083 | HP207450 | Human |
| Apoptosis / proliferation | *MKI67* | GAAAGAGTGGCAACCTGCCTTC | GCACCAAGTTTTACTACATCTGCC | NM_002417 | HP206104 | Human |
| Ferroptosis-related | *GPX4* | ACAAGAACGGCTGCGTGGTGAA | GCCACACACTTGTGGAGCTAGA | NM_002085 | HP225818 | Human |
| Ferroptosis-related | *SLC7A11* | TCCTGCTTTGGCTCCATGAACG | AGAGGAGTGTGCTTGCGGACAT | NM_014331 | HP210800 | Human |
| Ferroptosis-related | *ACSL4* | GCTATCTCCTCAGACACACCGA | AGGTGCTCCAACTCTGCCAGTA | NM_022977 | HP230899 | Human |
| Ferroptosis-related | *NCOA4* | GCTTGCTATTGGTGGAGTTCTCC | GCCATACCTCACGGCTTCTAAG | NM_005437 | HP208569 | Human |
| Ferroptosis-related | *NFE2L2* | CACATCCAGTCAGAAACCAGTGG | GGAATGTCTGCGCCAAAAGCTG | NM_006164 | HP209154 | Human |
| Reference gene | *GAPDH* | GTCTCCTCTGACTTCAACAGCG | ACCACCCTGTTGCTGTAGCCAA | NM_002046 | HP205798 | Human |
